# Supplementary material for: Effects of Thermal Stress on the Gut Microbiome of Juvenile Milkfish (Chanos chanos)
Source: Microorganisms. 2020 Dec 22;9(1):5. doi: 10.3390/microorganisms9010005 (PMC7822048; doi:10.3390/microorganisms9010005)
Supplement: Supplementary file 1 [file microorganisms-09-00005-s001.pdf]

Table S1: **Physiological biomarkers of milkfish exposed to thermal stress.** Temperature treatments: control temperature at 26°C (CT) and high temperature at 33°C (HT). Parameters were measured 0, 14, and 21 days after the temperature increase (Timepoint). Each treatment consisted of three independent tanks. Observations per tank and timepoint (N) used in the analysis of the relationship with the gut microbiome composition varied between 4 and 6.

**Treatment**	**Timepoint**	**Tank**	**N**	**LDH**	**IDH**	**Protein**	**Carbohydrates**	**Lipids**	**ETS**	**CEA**	**CAT**	**LPO**	**SOD**	**HSI**
CT	d0	T1	6	7399.276 ± 1101.947	34.581 ± 3.905	1147.761 ± 483.552	33.526 ± 19.177	136.755 ± 19.707	5.051 ± 0.384	264.342 ± 103.658	34018.804 ± 8305.695	35.902 ± 12.205	1.071 ± 0.32	77.298 ± 11.616
CT	d0	T3	5	8508.628 ± 1358.445	32.486 ± 5.753	1285.18 ± 571.926	36.014 ± 14.715	121.413 ± 26.849	5.038 ± 0.814	303.137 ± 144.996	41823.772 ± 8931.11	31.856 ± 14.95	1.081 ± 0.164	103.571 ± 14.597
CT	d0	T5	6	7943.338 ± 857.908	34.459 ± 4.827	1460.004 ± 547.122	22.15 ± 8.293	151.035 ± 31.04	5.514 ± 1.261	308.629 ± 132.919	39483.552 ± 9822.337	34.058 ± 16.699	1.065 ± 0.153	57.819 ± 12.302
CT	d14	T1	5	7266.646 ± 602.817	35.097 ± 6.448	1340.329 ± 511.502	66.138 ± 39.237	139.593 ± 36.699	5.511 ± 0.839	282.684 ± 94.964	34405.65 ± 11187.303	29.429 ± 11.081	1.446 ± 0.286	82.632 ± 38.284
CT	d14	T3	6	8567.201 ± 480.043	33.257 ± 2.684	1112.182 ± 431.073	44.409 ± 24.432	135.894 ± 24.783	5.391 ± 1.052	254.615 ± 122.812	38722.406 ± 9868.42	28.264 ± 9.228	1.093 ± 0.335	91.218 ± 29.192
CT	d14	T5	4	8721.729 ± 1304.927	40.08 ± 6.041	1195.956 ± 452.109	25.512 ± 15.107	143.963 ± 13.412	5.78 ± 0.537	238.797 ± 83.11	31190.948 ± 3149.675	36.691 ± 9.544	1.205 ± 0.194	87.193 ± 27.669
CT	d21	T1	4	7888.51 ± 717.648	29.087 ± 4.757	1300.116 ± 458.003	33.587 ± 12.972	111.183 ± 25.14	4.423 ± 0.51	320.852 ± 72.678	34720.186 ± 5936.183	34.796 ± 4.197	1.499 ± 0.159	90.475 ± 22.686
CT	d21	T3	5	8502.808 ± 771.527	34.407 ± 3.41	1340.983 ± 218.8	24.569 ± 9.057	129.927 ± 25.488	5.572 ± 0.567	273.886 ± 71.321	40692.676 ± 16155.382	25.005 ± 4.406	1.347 ± 0.251	86.117 ± 16.561
CT	d21	T5	6	8032.458 ± 919.152	34.63 ± 4.945	1169.041 ± 340.656	27.796 ± 18.658	127.951 ± 30.972	5.382 ± 0.552	248.104 ± 65.225	41164.041 ± 10771.974	23.186 ± 9.582	1.185 ± 0.153	62.396 ± 24.878
HT	d0	T2	5	8762.245 ± 712.216	41.185 ± 3.508	1149.988 ± 365.593	24.853 ± 12.052	124.934 ± 18.928	5.771 ± 0.725	223.059 ± 47.064	32106.228 ± 11949.74	39.568 ± 11.054	0.696 ± 0.118	140.819 ± 13.877
HT	d0	T4	6	8588.391 ± 893.049	46.839 ± 6.228	1567.738 ± 289.207	16.812 ± 8.533	127.101 ± 40.192	6.136 ± 0.849	283.3 ± 58.073	34492.298 ± 9095.673	63.471 ± 43.345	0.75 ± 0.156	162.196 ± 20.102
HT	d0	T6	5	8697.633 ± 899.509	49.465 ± 8.791	1223.806 ± 434.269	29.054 ± 19.165	112.665 ± 14.044	6.411 ± 1.001	210.544 ± 50.886	29324.305 ± 9801.002	44.574 ± 15.708	0.658 ± 0.133	163.74 ± 23.836
HT	d14	T2	6	9348.634 ± 1071.514	38.824 ± 6.311	1420.767 ± 465.365	41.127 ± 35.375	109.549 ± 16.828	5.647 ± 0.594	281.277 ± 85.333	30217.331 ± 2392.861	31.941 ± 17.031	0.926 ± 0.143	141.39 ± 25.095
HT	d14	T4	6	9107.059 ± 1079.84	46.063 ± 12.317	1295.893 ± 262.67	41.524 ± 21.208	111.311 ± 20.732	5.511 ± 0.958	269.174 ± 61.584	33761.616 ± 5070.728	35.933 ± 13.163	0.728 ± 0.105	155.393 ± 22.529
HT	d14	T6	6	8983.05 ± 1342.856	43.385 ± 7.12	1246.7 ± 200.029	35.893 ± 9.16	97.165 ± 21.469	5.94 ± 0.744	233.482 ± 28.801	33047.224 ± 7952.224	40.442 ± 7.474	0.789 ± 0.095	139.435 ± 33.663

Treatment	Timepoint	Tank	N	LDH	IDH	Protein	Carbohydrates	Lipids	ETS	CEA	CAT	LPO	SOD	HSI
HT	d21	T2	6	8765.859 ± 891.364	32.752 ± 4.848	1429.486 ± 343.947	32.911 ± 19.128	92.245 ± 18.501	4.784 ± 0.824	330.72 ± 78.849	33629.054 ± 3914.913	37.987 ± 18.301	1.076 ± 0.145	153.663 ± 26.922
HT	d21	T4	6	8209.01 ± 1012.872	34.461 ± 5.312	1200.235 ± 320.069	32.034 ± 13.027	92.9 ± 35.182	4.657 ± 0.759	289.479 ± 68.851	30425.309 ± 9522.033	26.031 ± 9.778	0.972 ± 0.219	139.933 ± 17.569
HT	d21	T6	5	9256.221 ± 2014.03	36.697 ± 8.058	1396.108 ± 561.721	25.491 ± 12.094	100.367 ± 21.577	4.847 ± 0.902	308.973 ± 97.058	32047.921 ± 5437.166	21.806 ± 8.656	1.037 ± 0.058	133.096 ± 48.285

LDH: activity of lactate dehydrogenase in muscle tissue [nmol (min mg protein)$^{-1}$]; IDH: activity of isocitrate dehydrogenase in muscle tissue [nmol (min mg protein)$^{-1}$]; Protein: total protein in muscle tissue [mJ (mg wet weight)$^{-1}$]; Carbohydrates: total carbohydrates in muscle tissue [mJ (mg wet weight)$^{-1}$]; Lipids: total lipids in muscle tissue [mJ (mg wet weight)$^{-1}$]; ETS: activity of electron transfer system in muscle tissue [mJ (h mg wet weight)$^{-1}$]; CEA: cellular energy allocation calculated according to Verslycke et al. (2004) as ratio of energy availability (sum of total protein, carbohydrates, and lipids in muscle tissue) and ETS; CAT: activity of catalase in liver tissue [nmol (min mg protein)$^{-1}$]; LPO: degree of lipid peroxidation in liver tissue [nmol TBARS (g wet weight)$^{-1}$] (TBARS: thiobarbituric acid reactive substances); SOD: activity of superoxide dismutase in liver tissue [U (mg protein)$^{-1}$]; HSI: hepatosomatic index, calculated as (liver weight/fish weight) *100.

SI table 2: **Differentially enriched OTUs between temperature treatments, sampling timepoints, and in correlation to physiological biomarkers.** Temperature treatments: control temperature at 26°C (CT) and high temperature at 33°C (HT). Sampling timepoints: 0, 14, and 21 days after the temperature increase. Each treatment consisted of three independent tanks. Observations per tank and timepoint (N) varied between 4 and 7. The differential OTU enrichment analysis was based on centered log ratio (clr)-transformed sequence counts of individual OTUs. The effects of treatment and timepoint were assessed using general linear mixed models with tank as random factor. Differentially enriched OTUs were defined based on Benjamini-Hochberg corrected p-values for each model term at a significance threshold of 0.05, followed by pairwise post hoc comparisons. Significantly different groups are indicated by lower case letters. Among the observed physiological biomarkers, only HSI (hepatosomatic index) showed a strong correlation with overall microbiome composition, and was therefore used in the differential OTU enrichment analysis. Moderate to strong correlations were defined at an absolute Pearson and Spearman correlation coefficient of at least 0.5, and their sign is provided in the table.

OTU	Genus	Treatment		Timepoint			Interaction						HSI
		CT	HT	d0	d14	d21	CT-d0	CT-d14	CT-21	HT-d0	HT-d14	HT-d21	
sq149	Acinetobacter			a	ab	b							
sq800	Alphaproteobacteria (uncl.)			a	a	b							
sq1305	Bradyrhizobium	b	a	a	a	b							pos
sq263	Brevibacterium			a	ab	b							
sq700	Burkholderiaceae (uncl.)	b	a	a	ab	b							pos
sq15	Catenococcus			a	ab	b							
sq20	Catenococcus			b	a	a							
sq46	Catenococcus			a	a	b							
sq1662	Catenococcus	b	a	a	a	b							pos
sq1	Cetobacterium	a	b	c	b	a	cd	b	a	d	cd	bc	neg
sq12	Cetobacterium	a	b	c	b	a							neg
sq23	Cetobacterium	a	b	c	b	a							neg
sq108	Cetobacterium	a	b	c	b	a							neg
sq127	Cetobacterium	a	b	c	b	a							neg
sq128	Cetobacterium	a	b	b	ab	a							
sq139	Cetobacterium	a	b	b	a	a							neg
sq140	Cetobacterium	a	b	c	b	a							neg
sq143	Cetobacterium	a	b	c	b	a							neg
sq147	Cetobacterium	a	b	c	b	a							neg
sq151	Cetobacterium	a	b	c	b	a							neg
sq154	Cetobacterium	a	b	c	b	a							neg
sq155	Cetobacterium	a	b	c	b	a							neg
sq157	Cetobacterium	a	b	c	b	a							neg
sq158	Cetobacterium	a	b	c	b	a							neg
sq159	Cetobacterium	a	b	b	a	a							neg

OTU	Genus	Treatment		Timepoint			Interaction						HSI
		**CT**	**HT**	**d0**	**d14**	**d21**	**CT-d0**	**CT-d14**	**CT-21**	**HT-d0**	**HT-d14**	**HT-d21**	
sq160	Cetobacterium	a	b	c	b	a							neg
sq162	Cetobacterium	a	b	b	a	a							neg
sq163	Cetobacterium	a	b	c	b	a							neg
sq164	Cetobacterium	a	b	b	a	a							neg
sq167	Cetobacterium	a	b	c	b	a							neg
sq168	Cetobacterium	a	b	b	a	a							neg
sq169	Cetobacterium	a	b	c	b	a							neg
sq173	Cetobacterium	a	b	c	b	a							neg
sq174	Cetobacterium	a	b	c	b	a							neg
sq176	Cetobacterium	a	b	c	b	a							neg
sq177	Cetobacterium	a	b	b	a	a							neg
sq180	Cetobacterium	a	b	b	a	a							neg
sq181	Cetobacterium	a	b	c	b	a							neg
sq182	Cetobacterium	a	b	c	b	a							neg
sq184	Cetobacterium	a	b	b	a	a							neg
sq186	Cetobacterium	a	b	b	a	a							neg
sq187	Cetobacterium	a	b	c	b	a							neg
sq188	Cetobacterium	a	b	c	b	a							neg
sq189	Cetobacterium	a	b	c	b	a							neg
sq190	Cetobacterium	a	b	b	a	a							neg
sq192	Cetobacterium	a	b	c	b	a							neg
sq193	Cetobacterium	a	b	c	b	a							neg
sq196	Cetobacterium	a	b	c	b	a							neg
sq197	Cetobacterium	a	b	c	b	a							neg
sq199	Cetobacterium	a	b	c	b	a							
sq203	Cetobacterium	a	b	c	b	a							neg
sq204	Cetobacterium	a	b	c	b	a							neg
sq207	Cetobacterium	a	b	c	b	a							neg
sq209	Cetobacterium	a	b	b	a	a							
sq212	Cetobacterium	a	b	c	b	a							neg
sq214	Cetobacterium	a	b	b	a	a							neg
sq220	Cetobacterium	a	b	c	b	a							neg
sq221	Cetobacterium	a	b	b	a	a							

OTU	Genus	Treatment		Timepoint			Interaction						HSI
		CT	HT	d0	d14	d21	CT-d0	CT-d14	CT-21	HT-d0	HT-d14	HT-d21	
sq225	Cetobacterium	a	b	b	a	a							neg
sq227	Cetobacterium	a	b	b	a	a							neg
sq228	Cetobacterium	a	b	b	a	a							neg
sq230	Cetobacterium	a	b	b	a	a							
sq231	Cetobacterium	a	b	b	a	a							neg
sq232	Cetobacterium	a	b	c	b	a							neg
sq234	Cetobacterium	a	b	b	a	a							neg
sq237	Cetobacterium	a	b	b	a	a							neg
sq241	Cetobacterium	a	b	b	a	a							
sq254	Cetobacterium	a	b	b	a	a							
sq259	Cetobacterium	a	b	b	b	a							neg
sq262	Cetobacterium	a	b	b	a	a							
sq306	Cetobacterium	a	b	b	a	a							
sq115	Corynebacterium 1	b	a										
sq53	Curvibacter	b	a										
sq34	Cutibacterium	b	a										
sq102	Cutibacterium	b	a										
sq77	Defluviimonas	b	a										
sq617	Dichotomicrobium	b	a										
sq494	Enterococcus			a	ab	b							
sq4	Enterovibrio			c	b	a	cd	ab	ab	bd	bd	ac	
sq7	Enterovibrio			c	b	a	cd	ab	ab	bd	bd	ac	
sq16	Enterovibrio			b	a	a							
sq25	Enterovibrio			b	b	a							
sq36	Enterovibrio			b	a	a							
sq48	Enterovibrio			b	b	a							
sq52	Enterovibrio			b	b	a							
sq93	Enterovibrio			b	ab	a							
sq279	Enterovibrio			c	b	a							
sq287	Epulopiscium						ab	ab	ab	b	a	a	
sq42	Francisella			a	b	b							
sq428	HIMB11	b	a	a	ab	b							pos
sq300	Lawsonella			a	a	b							

OTU	Genus	Treatment		Timepoint			Interaction						HSI
		CT	HT	d0	d14	d21	CT-d0	CT-d14	CT-21	HT-d0	HT-d14	HT-d21	
sq1104	Microbacteriaceae (uncl.)			a	ab	b							
sq250	Microbacterium	b	a	a	ab	b							
sq148	Ottowia			a	ab	b							
sq8	Romboutsia			b	ab	a							
sq91	Ruegeria	b	a										
sq707	Streptococcus			a	a	b							
sq84	Synechococcus CC9902	b	a	a	a	b							
sq296	Undibacterium	b	a	a	ab	b							
sq3	Vibrio			a	b	b							
sq17	Vibrio			b	a	a							
sq26	Vibrio			ab	b	a							
sq63	Vibrio	b	a										
sq65	Vibrio			a	b	b							
sq66	Vibrio			a	b	b							
sq109	Vibrio			b	a	ab							
sq223	Vibrio			a	b	b							
sq253	Vibrio			a	b	b							
sq299	Vibrio			a	b	b							
sq305	Vibrio			a	b	b							
sq312	Vibrio			a	b	b							
sq328	Vibrio			a	b	b							
sq336	Vibrio			a	b	c							
sq339	Vibrio			a	b	b							
sq348	Vibrio			a	b	b							
sq350	Vibrio			a	b	c							
sq360	Vibrio			a	b	c							
sq365	Vibrio			a	b	b							
sq367	Vibrio			a	b	b							pos
sq375	Vibrio			a	b	c							
sq384	Vibrio			a	b	b							
sq393	Vibrio			a	b	b							
sq402	Vibrio			a	b	c							
sq420	Vibrio			a	b	b							

OTU	Genus	Treatment		Timepoint			Interaction						HSI
		**CT**	**HT**	**d0**	**d14**	**d21**	**CT-d0**	**CT-d14**	**CT-21**	**HT-d0**	**HT-d14**	**HT-d21**	
sq504	Vibrio			a	b	c							
sq511	Vibrio			a	ab	b							
sq537	Vibrio			a	b	c							
sq549	Vibrio			a	b	c							pos
sq580	Vibrio			a	b	c							
sq674	Vibrio			a	b	c							pos
sq807	Vibrio			a	b	c							pos
sq928	Vibrio			a	b	c							
